# Evaluation of Serum Myostatin Concentration in Chronic Heart Failure with Preserved and Impaired Left Ventricular Ejection Fraction

**DOI:** 10.3390/jcm13061741

**Published:** 2024-03-18

**Authors:** Jan Bączek, Mirosław Charkiewicz, Agnieszka Kasiukiewicz, Anna Maria Witkowska, Łukasz Magnuszewski, Marta Bączek, Zyta Beata Wojszel

**Affiliations:** 1 Doctoral School, Medical University of Bialystok, 15-089 Bialystok, Poland; 2 Department of Cardiology, Hospital of the Ministry of Interior and Administration in Bialystok, Fabryczna 27, 15-471 Bialystok, Poland; miroslaw.charkiewicz@gmail.com; 3 Department of Geriatrics, Medical University of Bialystok, Fabryczna 27, 15-471 Bialystok, Poland; agnieszka.kasiukiewicz@umb.edu.pl (A.K.); zyta.wojszel@umb.edu.pl (Z.B.W.); 4 Department of Geriatrics, Hospital of the Ministry of Interior and Administration in Bialystok, Fabryczna 27, 15-471 Bialystok, Poland; 5 Department of Food Biotechnology, Medical University of Bialystok, Szpitalna 37, 15-295 Bialystok, Poland; 6 Faculty of Medicine with the Division of Dentistry and Division of Medical Education in English, Medical University of Bialystok, Kilińskiego 1, 15-089 Bialystok, Poland

**Keywords:** heart failure, left ventricular ejection fraction, sarcopenia, myostatin, muscle wasting, cardiac cachexia, biomarker, geriatrics, elderly

## Abstract

**Background:** Chronic heart failure (CHF) is a complex clinical syndrome associated with muscle wasting, which can progress to cardiac cachexia. Myostatin, a negative regulator of muscle growth, has been implicated in the pathophysiology of muscle wasting in CHF patients and suggested as a potential biomarker. The objective of this study was to investigate serum myostatin concentration in patients with CHF with preserved and reduced ejection fraction. **Methods:** The authors conducted a single-centre study comparing serum myostatin levels, functional and echocardiographic parameters, muscle mass, strength and function in patients with CHF to a control group without CHF. The study group was further divided into sub-groups with preserved and reduced or mildly reduced ejection fraction. **Results:** Results showed no significant differences in myostatin concentration between CHF patients and controls, and no correlation with sarcopenia or dynapenia. However, a higher myostatin concentration was found in patients with impaired systolic function (Me = 1675 pg/mL vs. Me—884.5 pg/mL; *p* = 0.007). A positive correlation between myostatin concentration and muscle mass (r = 0.27; *p* = 0.04), and functional parameters such as Norton (r = 0.35; *p* < 0.01), I-ADL (r = 0.28; *p* = 0.02) and Barthel scale (r = 0.27; *p* = 0.03) scores, was also observed. **Conclusions:** Myostatin appears to play a role in muscle wasting and its progression to cardiac cachexia in patients with impaired ejection fraction. Further research is needed to confirm these findings and explore myostatin’s potential as a biomarker for muscle loss and a target for pharmacotherapeutic agents in this population of patients.

## 1. Introduction

Sarcopenia, characterised by age-related loss of muscle quantity, quality, strength and performance, is a prevalent geriatric syndrome with a high impact on the hospitalisation risk, quality of life, disability and mortality of the elderly [1,2,3,4,5]. It is especially significant in the population of patients with chronic heart failure (CHF), where its prevalence is 20% higher and where it may ultimately progress to cardiac cachexia—a CHF complication associated with a high mortality rate [6]. Valid tools for early diagnosis are required in order to counteract the development and progression of sarcopenia with physical therapy and exercise, nutritional strategies and drugs being currently developed. Despite the new EWGSOP 2 consensus on the definition and diagnosis of sarcopenia, the credibility of the diagnostic process is still discussed [7], which highlights the need for a feasible biomarker of muscle loss.

Myostatin, or growth differentiation factor-8 (GDF-8), is a negative muscle growth regulator [8]. This protein functions as a myokine and is expressed mostly in skeletal muscles but also in cardiac muscle and adipose tissue [9,10]. For this reason, myostatin is seen as a potential biomarker of muscle wasting. Furthermore, it poses a probable foundation for novel drug agents in the therapy of sarcopenia and cachexia [11]. Although previous studies produced inconsistent results regarding the relationship between muscle mass and function and serum myostatin levels [12], higher serum myostatin concentration was found in patients with CHF. High circulating myostatin was also found to be a risk factor for death and rehospitalisation in CHF patients and was higher in patients with high N-terminal prohormone of brain natriuretic peptide (NT-proBNP) levels [13]. However, the relationship between serum myostatin abundance and left ventricular systolic function remains undetermined. Therefore, the aim of this study was to measure serum myostatin concentration in patients with preserved and impaired left ventricular systolic function and to assess the potential of myostatin as a biomarker of muscle wasting in those groups of patients.

## 2. Materials and Methods

### 2.1. Study Population

This observational study was conducted on patients admitted to the geriatric ward (sub-acute care) of the Hospital of the Ministry of Interior in Bialystok, Poland, between 1 March 2021 and 1 June 2022. Subjects were included if they were 65 or older and gave informed consent to participate in the study. Subjects with cancer cachexia, hemi- or paraparesis, neuromuscular disorders or renal failure during hemodialysis or unwilling or unable to give conscious consent were excluded from the study. After applying the inclusion and exclusion criteria, 67 patients were analysed in the study. The Ethics Committee approved the study at Medical University in Bialystok (approval number: APK.002.149.2021). All procedures performed in the study were in accordance with the ethical standards of the Medical University in Bialystok research committee and the Helsinki declaration and its later amendments.

### 2.2. Clinical Assessment

Clinical data on patients were acquired from medical charts and by direct history taking and physical examination. Data included demographic parameters, comorbidities (hypertension, orthostatic hypotension, diabetes, malignancy history, chronic obstructive pulmonary disease, asthma, past myocardial infarction, past stroke, ischaemic heart disease, arthritis, osteoporosis, chronic kidney disease, atrial fibrillation, past SARS-CoV-2 infection, valvular heart disease, peripheral arterial disease, Parkinson’s disease, dementia, depression, urinary incontinence), medication taken (angiotensin-converting enzyme inhibitors (ACE-I), angiotensin receptor blockers (ARB), angiotensin receptor-neprilysin inhibitors (ARNI), sodium-glucose co-transporter 2 (SGLT-2) inhibitors, beta-blockers, alpha-1-blockers, calcium channel blockers, loop diuretics, vitamin K antagonists, new oral anticoagulants, digoxin, corticosteroids, neuroleptics, acetylcholinesterase inhibitors, benzodiazepines, selective serotonin reuptake inhibitors, non-steroid anti-inflammatory drugs) and non-voluntary body mass loss. Multimorbidity was defined as 5 or more diseases diagnosed out of the 21 listed above, while polypharmacy was defined as 5 or more medications taken prior to admission to the hospital.

### 2.3. Anthropometric Measurements and Nutritional and Muscular Evaluation

Subjects’ height and weight were measured, and their body mass index (BMI) was calculated. Mid-arm circumference (MAC), calf circumference (CC), waist circumference and hip circumference were measured. Muscle strength was assessed by handgrip strength (HGS) measurement of the dominant hand using the DHD-1 dynamometer (SAEHAN, Masan, Republic of Korea). Muscle function was assessed in the Timed Up and Go (TUG) test and by gait speed measurement during the 4.57 m usual pace walk. Skeletal muscle mass (SMM) and skeletal muscle index (SMI = SMM/height^2^) were measured by bioimpedance analysis (BIA) using S10 Body Composition Analyzer (InBody, Seoul, Republic of Korea) [14]. Nutritional status was assessed using Mini Nutritional Assessment—Short Form (MNA-SF) [15] and Global Leadership Initiative on Malnutrition (GLIM) criteria [16]. Frailty was assessed using The 5-item FRAIL scale [17].

### 2.4. Questionnaires

All patients were asked to complete the SARC-F sarcopenia screening questionnaire [18]. EPIC Physical Activity Questionnaire (short version) was used to assess physical activity and energy expenditure [19,20]. Using a four-level physical activity index, subjects were divided into four groups (inactive, moderately inactive, moderately active and active) [21]. The functional, physical and mental status of patients was assessed in standard comprehensive geriatric assessment which included the 15-item Geriatric Depression Scale (GDS; 0–15 points) [22], Barthel index score (0–100 points) [23], the Duke Older American Resources and Services (OARS) I-ADL score (0–12 points) [24], pressure sore risk Norton Scale score (5–20 points) [25], Tinetti Performance Oriented Mobility Assessment (POMA, 0–28 points) [26] and Short Orientation Memory Concentration test (0–28 points) [27].

### 2.5. Transthoracic Echocardiography

Standard echocardiographic examinations using VIVID S70N and VIVID E95 (GE Healthcare, Wauwatosa, WI, USA) devices [28] were performed by a cardiologist experienced in echocardiography. Images of the long axis, the short axis and apical 4-chamber and 5-chamber projections were acquired. Left ventricle systolic and diastolic function was assessed according to the 2021 ESC Guidelines for the diagnosis and treatment of acute and chronic heart failure [29]. Left ventricular ejection fraction (LVEF) was calculated using modified Simpson’s rule [30].

### 2.6. Biochemical and Myostatin Measurements

Fasting venous blood was drawn into probes containing an anticoagulant. After being centrifuged for 15 min at 1000× *g*, serum samples were obtained. NT-proBNP, C-reactive protein (CRP) and creatinine concentration were measured using Cobas Pure analyser (Hitachi High-tech Croporation, Tokyo, Japan for Roche Diagnostics GmbH, Mannheim, Germany) with Roche reagents (Creatinine Jaffe REF. 08057532190 for immunoturbidimetric method with latex particles; CRP REF. 8057591190 for kinetic method with alcaic picrate and chromogene compensation; NT-proBNP REF. 09315284190 for biotin-streptavidin immunofluorescence method) [31]. Samples for myostatin measurements were stored at −80 °C until assayed. Serum myostatin concentration was measured using Human MSTN ELISA Kit REF. ORB546495 (Biorbyt, Cambridge, UK) in compliance with the manufacturer’s protocol [32]. Detection range was 31.2 pg/mL–2000 pg/mL with sensitivity < 10 pg/mL.

### 2.7. Study Parameters

All patients diagnosed with heart failure (HF) according to the 2021 ESC Guidelines for the diagnosis and treatment of acute and chronic heart failure [29] were assigned to a study group. The study group was further divided into two sub-groups. Patients with symptoms of CHF (I–IV NYHA class), LVEF ≥ 50% and objective evidence of structural and/or functional cardiac abnormalities, LV diastolic dysfunction or raised LV filling pressures and/or raised natriuretic peptides were diagnosed with heart failure with preserved ejection fraction (HFpEF) and assigned to sub-group 1 with preserved systolic function. Patients with symptoms of CHF and LVEF > 40% and <50% were diagnosed with heart failure with mildly reduced ejection fraction (HFmrEF), while patients with symptoms of CHF and LVEF ≤ 40% were diagnosed with heart failure with reduced ejection fraction (HFrEF). Patients with HFmrEF and HFrEF were analysed together, in sub-group 2 with impaired systolic function. Patients without heart failure (HF) were assigned to the control group.

**Figure 1:**
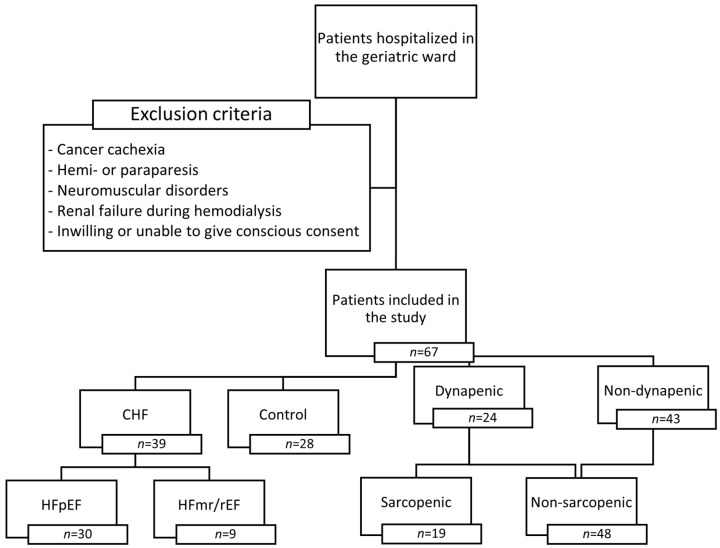
Flow chart of patient enrollment and study parameters. CHF—chronic heart failure; HFpEF—heart failure with preserved ejection fraction; HFmr/rEF—heart failure with mildly reduced or reduced ejection fraction.

Patients with HGS < 27 kg for males and <16 kg for females were qualified as dynapenic. Due to significant sarcopenia underdiagnosis using the 2019 European Working Group on Sarcopenia in Older People 2 (EWGSOP2) consensus criteria, low muscle mass was diagnosed according to the 2010 EWGSOP consensus [33]: dynapenic patients with SMI < 8.87 kg/m^2^ for males and <6.42 kg/m^2^ for females were classified as sarcopenic. Patient enrollment and study parameters are shown in Figure 1 flow chart.

### 2.8. Statistical Analysis

The IBM SPSS Version 18 Software suite (SPSS, Chicago, IL, USA) was used to analyse the data collected. The Kolmogorov–Smirnoff test was used to assess the normality of the distribution of the quantitative variables. Descriptive statistics for continuous variables were expressed as mean (M) and standard deviation (SD) or median (Me) and interquartile range (IQR) as appropriate. Categorical variables were expressed as frequency (N) and percentage (%). As appropriate, differences between groups were expressed using χ^2^ or Fisher’s exact test, Mann–Whitney or Student’s *t*-test. Missing values were omitted, and statistics were calculated for the adequately reduced groups. We performed Pearson’s correlation analysis on various study parameters including all subjects (*n* = 67). We performed a multivariable linear regression analysis to determine the association between myostatin and sarcopenia predictors with HF, including predictors with a *p*-value less than 0.1, excluding those highly correlated (to avoid multicollinearity) and controlling for the influence of age, gender and the number of chronic diseases. We reported ORs with 95% CIs and *p* values for each model parameter. Finally, we evaluated the statistical significance of the model with the Hosmer–Lemeshow goodness-of-fit C-statistics (significant *p*-value indicating an overall lack of fit). The results were considered statistically significant at two-tailed *p* < 0.05.

## 3. Results

Amongst 67 patients enrolled in the study, 39 (58%) were diagnosed with CHF and assigned to the study group, the characteristics of which are presented in Table 1. Supplementary information on the study and control groups can be found in Table A1 of the Appendix A. A total of 20 CHF patients (51.3%) presented swelling/oedema while 7 (17.9%) presented pulmonary crepitations. Five of them (12.8%) were classified as NYHA I, twelve (30.8%) as NYHA II, twenty-one (53.8%) as NYHA III and one (2.6%) as NYHA IV. CHF group patients were significantly older (Me = 84, IQR 78–87 years, versus Me = 79, IQR 74–82 years, in the control group, *p* = 0.004), were more often diagnosed with malnutrition or its risk (56.4% vs. 32.1%, *p* = 0.049), atrial fibrillation (66.7% vs. 17.9%, *p* < 0.001) and valvular heart disease (35.9% vs. 3.6%, *p* = 0.002) and were more often burdened with multimorbidity (76.9% vs. 28.6%, *p* < 0.001). They were treated with NOACs (56.4% vs. 7.1%, *p* < 0.001), loop diuretics (64.1% vs. 10.7%, *p* < 0.001) and MRA (41.0% vs. 3.6%, *p* = 0.002) significantly more frequently and took a larger number of medications (Me = 9, IQR 7–14, vs. Me = 6, IQR 4–8, *p* < 0.001). CHF patients also had significantly poorer renal function (eGFR Me = 47.8 mL/min, IQR 40.15–66.22 mL/min, vs. Me = 68.43 mL/min, IQR 56.52–81.84 mL/min, *p* = 0.002) and higher NT-proBNP levels (Me = 1250 pg/mL, IQR 560–2700 pg/mL, vs. Me = 182.5 pg/mL, IQR 102.5–360.5 pg/mL, *p* < 0.001). Regarding echocardiographic parameters, CH group patients had significantly lower EF, higher LAVI, higher medial E/E’ and higher lateral E’ velocity. CHF patients had significantly higher SARC-F scores and significantly lower scores on I-ADL, POMA and Norton scales. No other significant differences regarding medications taken, comorbidities or echocardiographic parameters were found.

Within the CHF group, 17 patients (45.9%) were dynapenic, and 14 subjects (40%) were classified as sarcopenic, with no significant difference compared to the control. Patients with CHF were more often burdened with frailty (53.8% vs. 35.7%); however, this difference was not statistically significant. Study group subjects were characterised by significantly worse muscle performance—longer TUG (Me = 22.16 s, IQR 16.64–28.89 s, vs. Me = 14.38 s, IQR 11–19.4 s, *p* = 0.003) and 4.57-metre walk test results (Me = 10 s, IQR 6.08–13.64 s, vs. Me = 5.46 s, IQR 4.56–9.10 s, *p* = 0.006, respectively) despite no significant difference in muscle strength or quantity. No significant difference in serum myostatin concentration or serum myostatin normalised to SMM was found between patients with CHF and the control group. Similarly, the study found no significant differences in serum myostatin concentrations between sarcopenic and non-sarcopenic or dynapenic and non-dynapenic patients.

**Figure 2:**
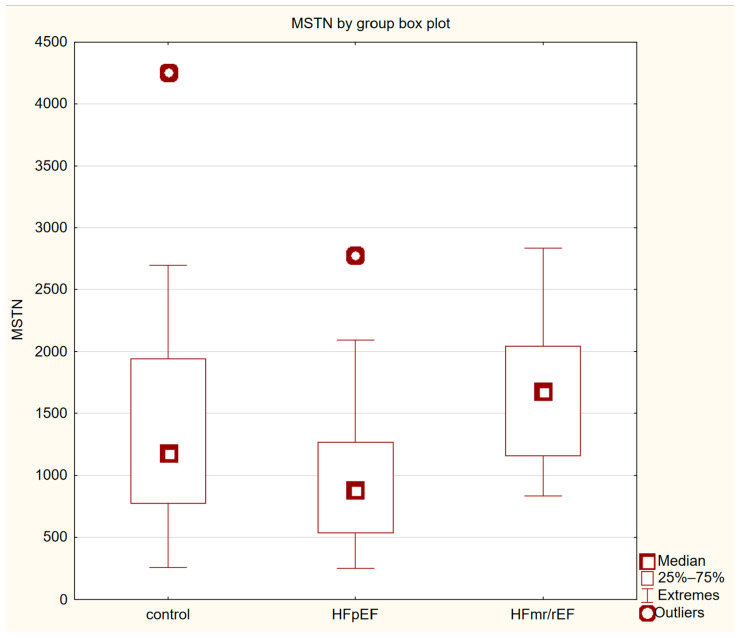
Myostatin concentration by group box plot.

Out of 39 study group patients, 30 (76.9%) were diagnosed with HFpEF, while 9 subjects (23.1%) were diagnosed with HFmrEF or HFrEF. The characteristics of these sub-groups can be found in Table 2. Supplementary information on HFpEF and HFmr/rEF groups can be found in Table A2 of the Appendix A. Further analysis of the study group revealed that patients with impaired LV systolic function had significantly higher serum myostatin concentrations (Me = 1675 pg/mL, IQR 1150–2294 pg/mL, vs. Me = 884.5 pg/mL, IQR 527.75–1284.75 pg/mL, *p* = 0.007) and serum myostatin normalised to SMM (Me = 70.38 pg/kg/mL, IQR 45.81–78.43 pg/mL/kg, vs. Me = 40.31 pg/mL/kg, IQR 21.58–52.75 pg/mL/kg, *p* = 0.02) in comparison to patients with preserved EF. Furthermore, we found that HFmr/rEF group patients had higher SMIs; however, this difference was on the verge of statistical significance. No other significant differences regarding anthropometric measurements, functional parameters, medications taken or comorbidities were found between those two subgroups. Myostatin concentration by group box plot can be found in Figure 2.

**Figure 3:**
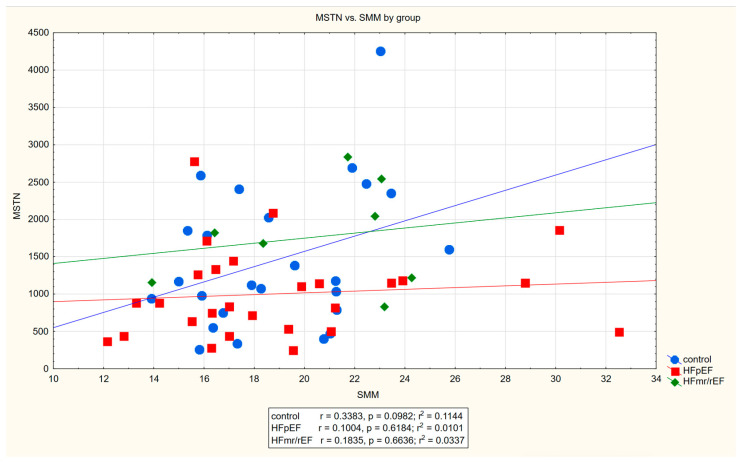
A scatter plot of myostatin concentration versus SMM.

Regarding myostatin association with muscle mass, strength and function, as well as other functional parameters, we found serum myostatin to be positively correlated with SMM (r = 0.27, *p* = 0.04) as well as Norton (r = 0.35, *p* < 0.01), I-ADL (r = 0.28, *p* = 0.02) and Barthel scale (r = 0.27, *p* = 0.03) scores. Furthermore, we found positive correlations of myostatin with SMI and a negative one with morbidity count, both on the verge of statistical significance. No substantial correlation with LVEF was found. The detailed results of Pearson’s correlation analysis can be found in Table 3. A scatter plot of myostatin concentration versus SMM can be found on Figure 3.

In order to perform multivariable linear regression, we selected parameters that reached or were close to statistical significance in previous analyses, which included 4MWT time, Barthel index score, I-ADL score, TUG time, SMM and HFmr/rEF as variables for the primary model. We performed backward stepwise linear regression until only variables that had a statistically significant influence on serum myostatin concentration remained. The fifth and final model presented impaired systolic LV function (patients with HFrEF or HFmrEF) as a positive predictor of serum myostatin concentration (standardised β-coefficient = 0.28, *p* = 0.05), while the 4MWT result emerged as a negative serum myostatin level predictor (standardised β-coefficient = −0.33, *p* = 0.02). Detailed regression results can be found in Table 4.

## 4. Discussion

In this study, we aimed to evaluate serum myostatin concentration in patients with chronic heart failure (CHF) with preserved and reduced ejection fraction in order to learn its role in the pathophysiology of cardiac cachexia and evaluate its potential as a biomarker of muscle wasting in these populations of patients. The need for such biomarkers is emphasised by reported difficulties with the diagnosis of sarcopenia, which we also experienced during this study [34]. We found a large difference in the number of sarcopenia cases amongst our study population when applying the latest 2019 EWGSOP2 consensus criteria in comparison with 2010 EWGSOP standards [33]. Therefore, we decided to use the earlier 2010 EWGSOP criteria.

The problem of significant underdiagnosis of sarcopenia when using the latest EWGSOP2 standard has been pointed out by various authors [34]. Van Ancum and al. found a significantly lower prevalence of sarcopenia in males according to EWGSOP2 criteria [7], while Wallengren et al. found a similar difference regardless of sex, with a higher confidence interval of the difference in the older population, suggesting a potential clinically significant difference between the standards in the older cohort [35]. Similarly, Fernandes et al. found EWGSOP2 criteria worse for predicting unfavourable outcomes and thus resulting in decreased sarcopenia prevalence [36]. These diagnostic difficulties highlight the necessity of a feasible biomarker of sarcopenia, which could aid the diagnostic process, facilitate early diagnosis and enable immediate nutritional and physiotherapeutic intervention. In the future, such a biomarker could also prove beneficial in initiating or monitoring casual sarcopenia treatment.

Myostatin is one of the proposed potential biomarkers of muscle wasting. Previous studies have produced inconsistent results regarding the relationship between serum myostatin levels and muscle mass and function [12,37,38,39,40,41,42,43,44,45,46,47,48,49]. Our study adds to the literature by focusing specifically on patients with CHF and comparing myostatin levels between patients with preserved and impaired left ventricular systolic function. The study results showed that patients with CHF had significantly worse muscle performance and higher SARC-F scores than the control group. However, there was no substantial difference in serum myostatin concentration between the two groups and no significant correlation between myostatin concentration and sarcopenia or dynapenia.

Furthermore, we found a significant difference in serum myostatin concentration between patients with preserved and impaired systolic function. Patients with impaired systolic function had significantly higher serum myostatin concentrations, despite no significant linear correlation of circulating myostatin level and LVEF. Interestingly, median skeletal muscle mass and SMI in this group were higher than in patients with preserved LVEF; however, this fact alone does not explain higher circulating myostatin abundance, since serum myostatin normalised to SMM was also significantly higher in patients with impaired systolic function. Ultimately, our findings were confirmed in multivariable linear regression analysis, where impaired systolic LV function emerged as a positive predictor of serum myostatin concentration, while the 4MWT result emerged as a negative serum myostatin level predictor. Our results confirm the findings of a previous study by Gruson et al. who found higher circulating myostatin levels in patients with CHF with LVEF < 35% with no significant linear correlation with LVEF [50].

The lack of significant difference in serum myostatin levels between the CHF and control groups is an interesting finding that contrasts with previous studies. Previous research has found higher serum myostatin levels in patients with CHF and even identified it as a risk factor for death and rehospitalisation, although some of them only studied HFrEF patients [13,50,51]. However, our study population was smaller and more homogeneous, which may explain the lack of significant difference. Another possible reason for discrepancies in our results with previous studies is the fact that myostatin is expressed not only in skeletal muscles but also in cardiac muscle and adipose tissue and has been found to be regulated by a variety of factors, such as physical activity and nutritional status, which could affect its usefulness as a singular biomarker [9,10,12]. It was recently suggested by Ladang et al. that, since the function of myostatin as a myokine is closely related to follistatin, a TGF-β ligand antagonist, the potential of these proteins as biomarkers could be greater if analysed together [52]. It is worth noting that despite the much lower median myostatin concentration in the HFpEF group in comparison to the control (884.5 vs. 1175.5 pg/mL), this disparity was statistically insignificant (*p* = 0.062). This difference could prove significant in a larger study on a higher number of patients and perhaps could be explained by lower muscle mass in HFpEF, as myostatin is produced in muscle tissue. Such discussion, however, requires more data and further analyses.

These findings may indicate the important role that myostatin could play in the pathophysiology of muscle wasting in heart failure and its progression to cardiac cachexia. Although myostatin may not be a feasible single biomarker of muscle wasting in CHF, our results suggest it might be viable in patients with HFmrEF and HFrEF, as well as a possible potent molecular grip point for future pharmacotherapeutic agents in this population of patients. Additionally, our study found a positive correlation between serum myostatin concentration and muscle mass, as well as with functional parameters such as Norton, I-ADL and Barthel scale scores. These findings suggest that myostatin is important in functional decline in elderly patients, regardless of their cardiac status.

It is worth noting that our study has several limitations. The most important limiting factor is its low sample size, which may have limited the statistical power of our analyses. Furthermore, we did not measure other potential biomarkers of muscle wasting or myostatin pathway metabolites, which could have provided a more comprehensive picture of muscle loss in our patient population. Finally, our study was conducted in a single center on geriatric patients, which may limit the generalisability of our findings to other populations. Therefore, the feasibility of myostatin as a biomarker should be examined in further studies on a larger number of patients, in tandem with other myokines.

## 5. Conclusions

In conclusion, our study examines a possible important factor in the pathophysiology of heart failure and the development of cardiac cachexia in patients with impaired left ventricular ejection fraction. Further research is needed to confirm our findings and determine the clinical utility of myostatin as a biomarker of muscle wasting and progression to cardiac cachexia in this patient population. Our study highlights diagnostic difficulties in the diagnosis of sarcopenia and the importance of identifying reliable biomarkers for monitoring muscle loss in patients with CHF, which could lead to early effective interventions to improve their quality of life and reduce their risk of hospitalisation and mortality.

## Figures and Tables

**Table 1:** Characteristics of study and control groups.

Parameter	Total	Control	Study (CHF)	*p* Value
No. (%) of patients	67 (100)	28 (41, 79)	39 (58, 21)	
Age, years, Me (IQR)	81 (77, 85)	79 (74, 82)	84 (78, 87)	0.004
Gender, male, n (%)	20 (29.9)	7 (25)	13 (33.3)	0.59
NYHA I, n (%)	-	-	5 (12.8)	-
NYHA II, n (%)	-	-	12 (30.8)	-
NYHA III, n (%)	-	-	21 (53.8)	-
NYHA IV, n (%)	-	-	1 (2.6)	-
Swelling/edema, n (%)	27 (40.3)	7 (25)	20 (51.3)	0.04
Pulmonary crepitations, n (%)	10 (14.9)	3 (10.7)	7 (17.9)	0.50
Dynapenia, n (%)	24 (38.1)	7 (26.9)	17 (45.9)	0.13
Sarcopenia, n (%)	19 (31.7)	5 (20)	14 (40)	0.16
Malnutrition or its risk, n (%)	31 (46.3)	9 (32.1)	22 (56.4)	0.049
Frail scale > 2 pts. (frail), n (%)	31 (46.3)	10 (35.7)	21 (53.8)	0.09
BMI, kg/m^2^, Me (IQR)	28.2 (24.8, 33)	28.4 (25.3, 33.2)	27.3 (24.8, 32.6)	0.62
Mid-arm circumference, cm, Me (IQR)	26 (24, 29)	27 (24, 29.3)	25 (23, 29)	0.19
Mid-calf circumference, cm, Me (IQR)	33.5 (30, 37.8)	33.5 (30, 37)	33.5 (31, 38)	0.56
Waist circumference, Me (IQR)	95.5 (85.3, 103)	95.5 (83, 104)	96.5 (86.8, 100.8)	0.69
Handgrip strength, kg, Me (IQR)	16.6 (12.7, 19.6)	17.7 (13.5, 23.7)	16.5 (12.3, 18.9)	0.20
TUG, s, Me (IQR)	19 (14, 26)	14.4 (11, 19.4)	22.2 (16.6, 28.9)	0.003
4MWT, s, Me (IQR)	8.1 (5, 12.3)	5.46 (4.6, 9.1)	10 (6.1, 13.6)	0.006
SMM, kg, Me (IQR)	23.65 (21.4, 27.2)	24.3 (21.7, 27.1)	23.3 (20.4, 27.7)	0.48
SMI, kg/m^2^, Me (IQR)	9.23 (8.5, 10.2)	9.36 (8.9, 10)	9.06 (8.3, 10.5)	0.46
SARC-F ≥ 4, n (%)	46 (68.7)	15 (53.6)	31 (79.5)	0.03
EPIC category—active and moderately active, n (%)	14 (21.2)	9 (32.1)	5 (13.2)	0.08
I-ADL, Me (IQR)	8 (6, 11)	9.5 (7, 12)	7 (4, 9)	0.001
Norton, Me (IQR)	18 (17, 19)	18 (18, 19)	17, (16, 18)	0.02
POMA, Me (IQR)	20 (16.3, 26.8)	23 (20, 28)	18 (15, 22)	<0.001
MSTN, pg/mL, Me (IQR)	1150 (719, 1719)	1175.5 (762.8, 1983)	1142 (639, 1445)	0.29
MSTN/SMM, pg/mL/kg, Me (IQR)	46.14 (29.5, 76.1)	47.26 (31.7, 83)	41.87 (29.1, 67)	0.28
CREA, mg/dL, Me (IQR)	0.92 (0.7, 1.2)	0.77 (0.7, 1)	1.06 (0.8, 1.2)	0.005
eGFR, mL/min, Me (IQR)	58.7 (43.6, 74)	68.43 (56.5, 81.8)	47.8 (40.2, 66.2)	0.002
CRP, mg/L, Me (IQR)	2.5 (1.2, 8.5)	1.7 (1.2, 8.2)	3.4 (1.2, 10.1)	0.48
NT-proBNP, pg/mL, Me (IQR)	560 (194, 1554)	182.5 (102.5, 360.5)	1250 (560, 2700)	<0.001
LVEF, %, Me (IQR)	62 (57, 65)	64 (69.3, 66)	60.25 (50, 65)	0.004
LVIDD, mm, Me (IQR)	47 (44, 51)	47 (43, 49)	48 (44, 51)	0.38
LAVI, mL/m^2^, Me (IQR)	52.84 (45.1, 69.6)	46.04 (34.6, 52.6)	62.24 (52.3, 76.7)	<0.001
Lateral E’ velocity, cm/s, Me (IQR)	10 (7, 12.8)	8 (7, 10)	11 (7, 14)	0.04
Medial E’ velocity, cm/s, Me (IQR)	7 (5.1, 10)	7 (5, 11)	7 (6, 9)	0.66
E/E’ lateral, Me (IQR)	8.86 (7, 10)	8.17 (6.7, 9.9)	9.35 (7.9, 10.8)	0.06
E/e’ medial, Me (IQR)	11.91 (9.3, 15)	10 (8.2, 12.6)	13 (10, 16.1)	0.002
E/A, Me (IQR)	0.85 (0.6, 1)	0.86 (0.6, 1)	0.84 (0.6, 1.51)	0.62
Morbidity count, n, Me (IQR)	5 (3, 7)	3 (2.3, 5.8)	6, (5, 8)	<0.001
Multimorbidity, n (%)	38 (56.7)	8 (28.6)	30 (76.9)	<0.001
Atrial fibrillation, n (%)	31 (46.3)	5 (17.9)	26 (66.7)	<0.001
Valvular heart disease, n (%)	15 (22.4)	1 (3.6)	14 (35.9)	0.002
Medication count, n, Me (IQR)	8 (5, 11)	6 (4, 8)	9 (7, 14)	<0.001
Polypharmacy, n (%)	55 (82.1)	20 (71.4)	35 (89.7)	0.10
Loop diuretics, n (%)	28 (41.8)	3 (10.7)	25 (64.1)	<0.001
MRA, n (%)	17 (25.4)	1 (3.6)	16 (41.0)	<0.001
NOAC, n (%)	24 (35.8)	2 (7.1)	22 (56.4)	<0.001

CHF—chronic heart failure; NYHA—New York Heart Association; BMI—body mass index; TUG—Timed Up and Go; 4MWT—4.57 m walk test; SMM—skeletal muscle mass; SMI—skeletal muscle index; I-ADL—instrumental activities of daily living; POMA—Performance Oriented Mobility Assessment; MSTN—myostatin; CREA—creatinine; eGFR—estimated glomerular filtration rate; CRP—C-reactive protein; NT-proBNP—N-terminal prohormone of brain natriuretic peptide; LVEF—left ventricular ejection fraction; LVIDD—left ventricular internal diastolic diameter; LAVI—left atrial volume index; MRA—mineralocorticoid receptor antagonists; NOAC—non-Vitamin K antagonist oral anticoagulants.

**Table 2:** Characteristics of HFpEF and HFmr/rEF groups.

Parameter	Total—HF	HFpEF	HFmr/rEF	*p* Value
No. (%) of patients	39 (100)	30 (76.9)	9 (23.1)	
Age, years, Me (IQR)	84 (78, 87)	84 (78, 86.25)	81 (76.5, 89.5)	0.71
Gender, male, n (%)	13 (33.3)	19 (30)	4 (44.4)	0.45
NYHA I, n (%)	5 (12.8)	4 (13.3)	1 (11.1)	0.81
NYHA II, n (%)	12 (30.8)	10 (33.3)	2 (22.2)	0.81
NYHA III, n (%)	21 (53.8)	15 (50)	6 (66.7)	0.81
NYHA IV, n (%)	1 (2.6)	1 (3.3)	0 (0)	0.81
Swelling/edema, n (%)	20 (51.3)	15 (50)	5 (55.6)	1.00
Pulmonary crepitations, n (%)	7 (17.9)	6 (20)	1 (11.1)	1.00
Dynapenia, n (%)	17 (45.9)	13 (44.8)	4 (50)	1.00
Sarcopenia, n (%)	14 (40)	13 (48.1)	1 (12.5)	0.11
Malnutrition or its risk, n (%)	22 (56.4)	16 (53.3)	6 (66.7)	0.70
Frail scale > 2 pts. (frail), n (%)	21 (53.8)	16 (53.3)	5 (55.6)	0.61
BMI, kg/m^2^, Me (IQR)	27.3 (24.8, 32.6)	27.3 (24.8, 32.6)	28.7 (24.6, 34.4)	0.83
Mid-arm circumference, cm, Me (IQR)	25 (23, 29)	25 (23, 29)	24 (22, 28)	0.42
Mid-calf circumference, cm, Me (IQR)	33.5 (31, 38)	34 (32, 38)	33 (27, 37)	0.40
Waist circumference, Me (IQR)	96.5 (86.8, 100.8)	98 (88.5, 101.5)	88 (86, 102)	0.60
Handgrip strength, kg, Me (IQR)	16.5 (12.3, 18.9)	18.9 (13.9, 25.7)	16.7 (14.2, 27.4)	0.90
TUG, s, Me (IQR)	22.16 (16.6, 28.9)	22.54 (15.9, 29.7)	20.86 (17.9, 31.6)	1.00
4MWT, s, Me (IQR)	10 (6.1, 13.6)	9.34 (5.7, 13.5)	12.21 (7.3, 18)	0.33
SMM, kg, Me (IQR)	23.3 (20.4, 27.7)	22.1 (20.2, 26.9)	27.45 (23.7, 30)	0.08
SMI, kg/m^2^, Me (IQR)	9.06 (8.3, 10.5)	8.88 (8.2, 0.82)	10.14 (9.4, 11.23)	0.06
SARC-F ≥ 4, n (%)	31 (79.5)	23 (76.7)	8 (88.9)	0.65
EPIC category—active and moderately active, n (%)	5 (13.2)	4 (13.8)	1 (11.1)	1.00
I-ADL, Me (IQR)	7 (4, 9)	6 (4, 8)	7 (5, 10)	0.35
Norton, Me (IQR)	17 (16, 18)	17 (16, 18)	18 (16.5, 19.5)	0.38
POMA, Me (IQR)	18 (15, 22)	18 (15.3, 23)	16 (13, 22)	0.34
MSTN, pg/mL, Me (IQR)	1142 (639, 1445)	884.5 (527.8, 1284.8)	1675 (1150, 2294)	0.007
MSTN/SMM, pg/mL/kg, Me (IQR)	41.87 (29.06, 67)	40.31 (21.6, 52.8)	70.38 (45.8, 78.4)	0.02
CREA, mg/dL, Me (IQR)	1.06 (0.8, 1.2)	1.07 (0.7, 1.2)	0.8 (0.4, 1.8)	0.68
eGFR, mL/min, Me (IQR)	47.8 (40.2, 66.2)	49.87 (39.1, 67.8)	47.16 (44.3, 66)	0.91
CRP, mg/L, Me (IQR)	3.4 (1.2, 10.1)	4.05 (1.7, 11.8)	1.4 (0.7, 8.7)	0.11
NT-proBNP, pg/mL, Me (IQR)	1250 (560, 2700)	1205 (554.5, 2676.7)	1429 (466.5, 6767.5)	0.63
LVEF, %, Me (IQR)	60.25 (50, 65)	62 (57, 65)	40 (33.5, 43.5)	<0.001
LVIDD, mm, Me (IQR)	48 (44, 51)	46.5 (44, 50.25)	51 (47, 56)	0.03
LAVI, mL/m^2^, Me (IQR)	62.24 (52.3, 76.7)	62.23 (52.3, 77.4)	61.76 (49.8, 76.2)	0.84
Lateral E’ velocity, cm/s, Me (IQR)	11 (7, 14)	12 (8.25, 15)	8 (7, 9)	0.06
Medial E’ velocity, cm/s, Me (IQR)	7 (6, 9)	8 (6, 10)	6.5 (4, 7.8)	0.05
E/E’ lateral, Me (IQR)	9.35 (7.9, 10.8)	9.3 (7.8, 10)	10 (7.8, 12)	0.35
E/e’ medial, Me (IQR)	13 (10, 16.1)	13 (10.6, 15.1)	13.13 (8, 28.3)	0.79
E/A, Me (IQR)	0.84 (0.6, 1.5)	1 (0.6, 1.5)	0.8 (0.4, 1.8)	0.44
Morbidity count, n, Me (IQR)	6, (5, 8)	6 (4, 7)	7 (5.5, 8)	0.15
Multimorbidity, n (%)	30 (76.9)	22 (73.3)	8 (88.9)	0.65
Atrial fibrillation, n (%)	26 (66.7)	19 (63.3)	7 (77.8)	0.69
Valvular heart disease, n (%)	14 (35.9)	11 (36.7)	3 (33.3)	1.00
Medication count, n, Me (IQR)	9 (7, 14)	10 (6.8, 14.5)	9 (7.5, 12.5)	0.81
Polypharmacy, n (%)	35 (89.7)	27 (90)	8 (88.9)	1.00
Loop diuretics, n (%)	25 (64.1)	19 (63.3)	6 (66.7)	1.00
MRA, n (%)	16 (41.0)	14 (46.7)	2 (22.2)	0.26
NOAC, n (%)	22 (56.4)	17 (56.7)	5 (55.6)	1.00

NYHA—New York Heart Association; BMI—body mass index; TUG—Timed Up and Go; 4MWT—4.57 m walk test; SMM—skeletal muscle mass; SMI—skeletal muscle index; I-ADL—instrumental activities of daily living; POMA—Performance Oriented Mobility Assessment; MSTN—myostatin; CREA—creatinine; eGFR—estimated glomerular filtration rate; CRP—C-reactive protein; NT-proBNP—N-terminal prohormone of brain natriuretic peptide; LVEF—left ventricular ejection fraction; LVIDD—left ventricular internal diastolic diameter; LAVI—left atrial volume index; MRA—mineralocorticoid receptor antagonists; NOAC—non-Vitamin K antagonist oral anticoagulants.

**Table 3:** Pearson’s correlations of serum myostatin concentration with other parameters (*n* = 67).

	Age	Mass	BMI	LVEF	NT-proBNP	Morbidity count	SARC-F	NYHA
r	−0.03	0.03	−0.04	−0.07	−0.24	0.02	−0.15	−0.18
*p*-value	0.84	0.80	0.77	0.59	0.05	0.85	0.22	0.16
	HGS	TUG	4MWT	SMM	SMI	Barthel index	I-ADL scale	Norton scale
r	0.22	−0.24	−0.24	0.27	0.25	0.27	0.28	0.35
*p*-value	0.08	0.08	0.07	0.04	0.06	0.03	0.02	<0.01

NYHA—New York Heart Association; BMI—body mass index; TUG—Timed Up and Go; 4MWT—4.57 m walk test; SMM—skeletal muscle mass; SMI—skeletal muscle index; I-ADL—instrumental activities of daily living; NT-proBNP—N-terminal prohormone of brain natriuretic peptide; LVEF—left ventricular ejection fraction; HGS—handgrip strength.

**Table 4:** Multivariable linear regression analysis for serum myostatin (*n* = 67).

Model	Predictors	β-Coefficient	95% CI		Standardised β-Coefficient	*p*-Value
Primary model r^2^ = 0.194, F = 1.643	(const.)	649.77	−1576.10	2875.65		0.56
	4MWT	−25.60	−108.94	57.74	−0.15	0.54
	BARTHEL	3.60	−11.79	18.99	0.08	0.64
	I-ADL	29.87	−60.05	119.78	0.12	0.51
	TUG	−6.12	−44.19	31.94	−0.08	0.75
	SMM	19.16	−34.44	72.76	0.11	0.48
	HFmr/rEF	709.60	−117.52	1536.71	0.26	0.10
Final model r^2^ = 0.158, F = 4.213	(const.)	1815.09	1328.37	2301.82		<0.01
	4MWT	−56.34	−104.24	−8.45	−0.33	0.02
	HFmr/rEF	784.05	7.60	1560.50	0.28	0.05

TUG—Timed Up and Go; 4MWT—4.57 m walk test; SMM—skeletal muscle mass; I-ADL—instrumental activities of daily living; HFmr/rEF—heart failure with mildly reduced or reduced ejection fraction.

## Data Availability

The datasets presented in this article are not readily available due to technical and tie limitations. Requests to access the datasets should be directed to the corresponding author.

## Appendix A

**Table A1:** Full characteristics of study and control groups.

Parameter	Total	Control	Study (CHF)	*p* Value
No. (%) of patients	67 (100)	28 (41, 79)	39 (58, 21)	
Age, years, Me (IQR)	81 (77, 85)	79 (74, 82)	84 (78, 87)	0.004
Gender, male, n (%)	20 (29.9)	7 (25)	13 (33.3)	0.59
NYHA I, n (%)	-	-	5 (12.8)	-
NYHA II, n (%)	-	-	12 (30.8)	-
NYHA III, n (%)	-	-	21 (53.8)	-
NYHA IV, n (%)	-	-	1 (2.6)	-
Swelling/Oedema, n (%)	27 (40.3)	7 (25)	20 (51.3)	0.044
Pulmonary crepitations, n (%)	10 (14.9)	3 (10.7)	7 (17.9)	0.50
Dynapenia, n (%)	28 (44.4)	9 (34.6)	19 (51.4)	0.21
Sarcopenia, n (%)	19 (31.7)	5 (20)	14 (40)	0.16
Malnutrition or its risk, n (%)	31 (46.3)	9 (32.1)	22 (56.4)	0.049
Clinical frailty scale 0 pts. (robust), n (%)	8 (11.9)	6 (21.4)	2 (5.1)	0.09
Clinical frailty scale 1–2 pts. (pre-frail), n (%)	28 (41.8)	12 (42.9)	16 (41.0)	0.09
Clinical frailty scale > 2 pts. (frail), n (%)	31 (46.3)	10 (35.7)	21 (53.8)	0.09
BMI, kg/m^2^, Me (IQR)	28.2 (24.8, 33)	28.4 (25.3, 33.2)	27.3 (24.8, 32.6)	0.62
Mid-arm circumference, cm, Me (IQR)	26 (24, 29)	27 (24, 29.3)	25 (23, 29)	0.19
Mid-calf circumference, cm, Me (IQR)	33.5 (30, 37.8)	33.5 (30, 37)	33.5 (31, 38)	0.56
Waist circumference, Me (IQR)	95.5 (85.3, 103)	95.5 (83, 104)	96.5 (86.8, 100.8)	0.69
Right handgrip strength, kg, Me (IQR)	17 (13, 26.1)	18.5 (13.7, 27.3)	15.8 (12.9, 25)	0.56
Left handgrip strength, kg, Me (IQR)	16.5 (12, 24.8)	16.5 (11.9, 25.1)	16.5 (12, 23.1)	0.88
TUG, s, Me (IQR)	19 (14, 26)	14.4 (11, 19.4)	22.2 (16.6, 28.9)	0.003
4MWT, s, Me (IQR)	8.1 (5, 12.3)	5.46 (4.6, 9.1)	10 (6.1, 13.6)	0.006
SMM, kg, Me (IQR)	23.65 (21.4, 27.2)	24.3 (21.7, 27.1)	23.3 (20.4, 27.7)	0.48
SMI, kg/m^2^, Me (IQR)	9.23 (8.5, 10.2)	9.36 (8.9, 10)	9.06 (8.3, 10.5)	0.46
ALM, kg, Me (IQR)	18.32 (16.1, 21.6)	18.27 (16, 21.3)	18.37 (16.1, 22.8)	0.97
ALM/height^2^, kg/m^2^, Me (IQR)	7.24 (6.5, 8.1)	7.10 (6.5, 8)	7.35 (6.5, 8.2)	0.60
SARC-F ≥ 4, n (%)	46 (68.7)	15 (53.6)	31 (79.5)	0.033
EPIC category—inactive, n (%)	34 (51.5)	20 (52.6)	14 (50)	0.07
EPIC category—moderately inactive, n (%)	18 (27.3)	5 (17.9)	13 (34.2)	0.07
EPIC category—moderately active, n (%)	7 (10.6)	3 (10.7)	4 (10.5)	0.07
EPIC category—active, n (%)	7 (10.6)	6 (21.4)	1 (2.6)	0.07
EPIC category—active and moderately active, n (%)	14 (21.2)	9 (32.1)	5 (13.2)	0.08
Falls during last year, n (%)	36 (53.7)	13 (46.4)	23 (59)	0.33
MNA-Sf 12–14, n (%)	36 (53.7)	19 (67.9)	17 (43.6)	0.07
MNA-Sf 8–11, n (%)	25 (37.3)	6 (21.4)	19 (48.7)	0.07
MNA-Sf 0–7, n (%)	6 (9)	3 (10.7)	3 (7.7)	0.07
MNA-Sf, Me (IQR)	12 (10, 13)	95 (11, 13.8)	11 (10, 13)	0.14
Barthel index, Me (IQR)	90 (75, 95)	95 (77.5, 100)	90 (70, 95)	0.12
I-ADL, Me (IQR)	8 (6, 11)	9.5 (7, 12)	7 (4, 9)	0.001
Norton, Me (IQR)	18 (17, 19)	18 (18, 19)	17, (16, 18)	0.020
GDS, Me (IQR)	5 (3, 8)	4.5 (2, 8)	5 (3, 9)	0.25
Blessed, Me (IQR)	4 (2, 10)	4 (2, 10)	6 (2, 12)	0.19
POMA, Me (IQR)	20 (16.3, 26.8)	23 (20, 28)	18 (15, 22)	<0.001
MSTN, pg/mL, Me (IQR)	1150 (719, 1719)	1175.5 (762.8, 1983)	1142 (639, 1445)	0.29
MSTN/SMM, pg/mL/kg, Me (IQR)	46.14 (29.5, 76.1)	47.26 (31.7, 83)	41.87 (29.1, 67)	0.28
CREA, mg/dL, Me (IQR)	0.92 (0.7, 1.2)	0.77 (0.7, 1)	1.06 (0.8, 1.2)	0.005
eGFR, mL/min, Me (IQR)	58.7 (43.6, 74)	68.43 (56.5, 81.8)	47.8 (40.2, 66.2)	0.002
CRP, mg/L, Me (IQR)	2.5 (1.2, 8.5)	1.7 (1.2, 8.2)	3.4 (1.2, 10.1)	0.48
NT-proBNP, pg/mL, Me (IQR)	560 (194, 1554)	182.5 (102.5, 360.5)	1250 (560, 2700)	<0.001
LVEF, %, Me (IQR)	62 (57, 65)	64 (69.3, 66)	60.25 (50, 65)	0.004
LVIDD, mm, Me (IQR)	47 (44, 51)	47 (43, 49)	48 (44, 51)	0.38
LAVI, mL/m^2^, Me (IQR)	52.84, (45.1, 69.6)	46.04 (34.6, 52.6)	62.24 (52.3, 76.7)	<0.001
LVMI, g/m^2^, Me (IQR)	104.24 (89.1, 115.4)	101.63 (76.6, 114.1)	104.76 (95.9, 116.9)	0.13
Lateral E’ velocity, cm/s, Me (IQR)	10 (7, 12.8)	8 (7, 10)	11 (7, 14)	0.039
Medial E’ velocity, cm/s, Me (IQR)	7 (5.1, 10)	7 (5, 11)	7 (6, 9)	0.66
E/E’ lateral, Me (IQR)	8.86 (7, 10)	8.17 (6.7, 9.9)	9.35 (7.9, 10.8)	0.06
E/e’ medial, Me (IQR)	11.91 (9.3, 15)	10 (8.2, 12.6)	13 (10, 16.1)	0.002
E/A, Me (IQR)	0.85 (0.6, 1)	0.86 (0.6, 1)	0.84 (0.6, 1.51)	0.62
Morbidity count, n, Me (IQR)	5 (3, 7)	3 (2.3, 5.8)	6, (5, 8)	<0.001
Multimorbidity, n (%)	38 (56.7)	8 (28.6)	30 (76.9)	<0.001
Hypertension, n (%)	54 (80.6)	24 (85.7)	30 (76.9)	0.53
Orthostatic hypotension, n (%)	20 (29.9)	8 (28.6)	12 (30.8)	1.00
Diabetes mellitus, n (%)	21 (31.3)	8 (28.6)	13 (33.3)	0.79
Carcinoma history, n (%)	2 (3)	0 (0)	2 (5.1)	0.51
Chronic obstructive pulmonary disease, n (%)	12 (17.9)	2 (7.1)	10 (25.6)	0.06
Myocardial infarction history, n (%)	8 (11.9)	2 (7.1)	6 (15.4)	0.45
Ischemic heart disease, n (%)	19 (28.4)	6 (21.4)	13 (33.3)	0.41
Asthma, n (%)	5 (7.5)	2 (7.1)	3 (7.7)	1.00
Arthritis, n (%)	5 (7.5)	2 (7.1)	3 (7.7)	1.00
Osteoporosis, n (%)	20 (29.9)	8 (28.6)	12 (30.8)	1.00
Stroke history, n (%)	6 (9)	2 (7.1)	4 (10.3)	1.00
Chronic kidney disease, n (%)	15 (22.4)	3 (10.7)	12 (30.8)	0.08
Atrial fibrillation, n (%)	31 (46.3)	5 (17.9)	26 (66.7)	<0.001
COVID-19 history, n (%)	8 (11.9)	6 (21.4)	2 (5.1)	0.06
Peripheral arterial disease, n (%)	3 (4.5)	0 (0)	3 (7.7)	0.26
Valvular heart disease, n (%)	15 (22.4)	1 (3.6)	14 (35.9)	0.002
Parkinson’s disease, n (%)	5 (7.5)	1 (3.6)	4 (10.3)	0.39
Dementia, n (%)	24 (35.8)	8 (28.6)	16 (41)	0.32
Depression, n (%)	37 (55.3)	18 (64.3)	19 (48.7)	0.23
Urinary incontinence, n (%)	1 (1.5)	0(0)	1 (2.6)	1.00
Medication count, n, Me (IQR)	8 (5, 11)	6 (4, 8)	9 (7, 14)	<0.001
Polypharmacy, n (%)	55 (82.1)	20 (71.4)	35 (89.7)	0.10
ACE-I, n (%)	25 (37.3)	11 (39.3)	14 (35.9)	0.80
ARB, n (%)	16 (23.9)	8 (28.6)	8 (20.5)	0.56
Beta-blockers, n (%)	46 (68.7)	18 (64.3)	28 (71.8)	0.60
Alpha1-blockers, n (%)	10 (14.9)	3 (10.7)	7 (17.9)	0.50
Loop diuretics, n (%)	28 (41.8)	3 (10.7)	25 (64.1)	<0.001
MRA, n (%)	17 (25.4)	1 (3.6)	16 (41.0)	<0.001
Thiazide diuretics, n (%)	9 (13.4)	4 (14.3)	5 (12.8)	1.00
Calcium channel blockers, n (%)	18 (26.9)	7 (25)	11 (28.2)	1.00
Statin, n (%)	20 (29.9)	9 (23.1)	11 (28.2)	0.79
Fibrate, n (%)	1 (1.5)	0 (0)	1 (2.6)	1.00
VKA, n (%)	4 (6)	1 (3.6)	3 (7.7)	0.64
NOAC, n (%)	24 (35.8)	2 (7.1)	22 (56.4)	<0.001
Digoxin, n (%)	6 (9)	1 (3.6)	5 (12.8)	0.39
Steroids, n (%)	3 (4.5)	2 (7.1)	1 (2.6)	0.57
Neuroleptics, n (%)	9 (13.4)	4 (14.3)	5 (12.8)	1.00
Memantine, n (%)	2 (3)	1 (3.6)	1 (2.6)	1.00
Donepezil, n (%)	3 (4.5)	0 (0)	3 (7.7)	0.26
Rivastigmine, n (%)	2 (3)	1 (3.6)	1 (2.6)	1.00
Benzodiazepine, n (%)	7 (10.5)	2 (7.1)	5 (12.8)	0.69
SSRI, n (%)	22 (32.8)	6 (21.4)	16 (41.0)	0.12
NSAID, n (%)	11 (16.4)	5 (17.9)	6 (15.4)	1.00

CHF—chronic heart failure; NYHA—New York Heart Association; BMI—body mass index; TUG—Timed Up and Go; 4MWT—4.57 m walk test; SMM—skeletal muscle mass; SMI—skeletal muscle index; ALM—appendicular lean mass; MNA-Sf—Mini Nutritional Assessment—Short Form; I-ADL—instrumental activities of daily living; GDS—Geriatric Depression Scale; POMA—Performance Oriented Mobility Assessment; MSTN—myostatin; CREA—creatinine; eGFR—estimated glomerular filtration rate; CRP—C-reactive protein; NT-proBNP—N-terminal prohormone of brain natriuretic peptide; LVEF—left ventricular ejection fraction; LVIDD—left ventricular internal diastolic diameter; LAVI—left atrial volume index; LVMI—left ventricular mass index; ACE-I—angiotensin-converting enzyme inhibitors; ARB—angiotensin receptor blockers; MRA—mineralocorticoid receptor antagonists; VKA—vitamin K antagonists; NOAC—non-Vitamin K antagonist oral anticoagulants; SSRI—selective serotonin reuptake inhibitors; NSAID—non-steroidal anti-inflammatory drugs.

**Table A2:** Full characteristics of HFpEF and HFmr/rEF groups.

Parameter	Total—HF	HFpEF	HFmr/rEF	*p* Value
No. (%) of patients	39 (100)	30 (76.9)	9 (23.1)	
Age, years, Me (IQR)	84 (78, 87)	84 (78, 86.25)	81 (76.5, 89.5)	0.71
Gender, male, n (%)	13 (33.3)	19 (30)	4 (44.4)	0.45
NYHA I, n (%)	5 (12.8)	4 (13.3)	1 (11.1)	0.81
NYHA II, n (%)	12 (30.8)	10 (33.3)	2 (22.2)	0.81
NYHA III, n (%)	21 (53.8)	15 (50)	6 (66.7)	0.81
NYHA IV, n (%)	1 (2.6)	1 (3.3)	0 (0)	0.81
Swelling/edema, n (%)	20 (51.3)	15 (50)	5 (55.6)	1.00
Pulmonary crepitations, n (%)	7 (17.9)	6 (20)	1 (11.1)	1.00
Dynapenia, n (%)	19 (51.4)	14 (48.3)	5 (62.5)	0.69
Sarcopenia, n (%)	14 (40)	13 (48.1)	1 (12.5)	0.11
Malnutrition or its risk, n (%)	22 (56.4)	16 (53.3)	6 (66.7)	0.70
Clinical frailty scale 0 pts. (robust), n (%)	2 (5.1)	1 (3.3)	1 (11.1)	0.61
Clinical frailty scale 1–2 pts. (pre-frail), n (%)	16 (41.0)	13 (43.3)	3 (33.3)	0.61
Clinical frailty scale > 2 pts. (frail), n (%)	21 (53.8)	16 (53.3)	5 (55.6)	0.61
BMI, kg/m^2^, Me (IQR)	27.3 (24.8, 32.6)	27.3 (24.8, 32.6)	28.7 (24.6, 34.4)	0.83
Mid-arm circumference, cm, Me (IQR)	25 (23, 29)	25 (23, 29)	24 (22, 28)	0.42
Mid-calf circumference, cm, Me (IQR)	33.5 (31, 38)	34 (32, 38)	33 (27, 37)	0.40
Waist circumference, Me (IQR)	96.5 (86.8, 100.8)	98 (88.5, 101.5)	88 (86, 102)	0.60
Right handgrip strength, kg, Me (IQR)	15.8 (12.9, 25)	16.3 (12.9, 25)	15.25 (13, 26)	0.79
Left handgrip strength, kg, Me (IQR)	16.5 (12, 23.1)	16.5 (12.2, 23.1)	14.3 (11.1, 26.9)	0.55
TUG, s, Me (IQR)	22.16 (16.6, 28.9)	22.54 (15.9, 29.7)	20.86 (17.9, 31.6)	1.00
4MWT, s, Me (IQR)	10 (6.1, 13.6)	9.34 (5.7, 13.5)	12.21 (7.3, 18)	0.33
SMM, kg, Me (IQR)	23.3 (20.4, 27.7)	22.1 (20.2, 26.9)	27.45 (23.7, 30)	0.08
SMI, kg/m^2^, Me (IQR)	9.06 (8.3, 10.5)	8.88 (8.2, 0.82)	10.14 (9.4, 11.23)	0.056
ALM, kg, Me (IQR)	18.37 (16.1, 22.8)	17.15 (15.8, 21)	22.27 (16.9, 23.2)	0.22
ALM/height^2^, kg/m^2^, Me (IQR)	7.35 (6.5, 8.2)	7.25 (6.5, 7.7)	8.15 (6.7, 9.2)	0.25
MSTN, pg/mL, Me (IQR)	1142 (639, 1445)	884.5 (527.8, 1284.8)	1675 (1150, 2294)	0.007
MSTN/SMM, pg/mL/kg, Me (IQR)	41.87 (29.06, 67)	40.31 (21.6, 52.8)	70.38 (45.8, 78.4)	0.024
CREA, mg/dL, Me (IQR)	1.06 (0.8, 1.2)	1.07 (0.7, 1.2)	0.8 (0.4, 1.8)	0.68
eGFR, mL/min, Me (IQR)	47.8 (40.2, 66.2)	49.87 (39.1, 67.8)	47.16 (44.3, 66)	0.91
CRP, mg/L, Me (IQR)	3.4 (1.2, 10.1)	4.05 (1.7, 11.8)	1.4 (0.7, 8.7)	0.11
NT-proBNP, pg/mL, Me (IQR)	1250 (560, 2700)	1205 (554.5, 2676.7)	1429 (466.5, 6767.5)	0.63
LVEF, %, Me (IQR)	60.25 (50, 65)	62 (57, 65)	40 (33.5, 43.5)	<0.001
LVIDD, mm, Me (IQR)	48 (44, 51)	46.5 (44, 50.25)	51 (47, 56)	0.027
LAVI, mL/m^2^, Me (IQR)	62.24 (52.3, 76.7)	62.23 (52.3, 77.4)	61.76 (49.8, 76.2)	0.84
LVMI, g/m^2^, Me (IQR)	104.76 (95.9, 116.9)	103.71 (95.3, 116.5)	108.56 (99.5, 136.5)	0.27
Lateral E’ velocity, cm/s, Me (IQR)	11 (7, 14)	12 (8.25, 15)	8 (7, 9)	0.06
Medial E’ velocity, cm/s, Me (IQR)	7 (6, 9)	8 (6, 10)	6.5 (4, 7.8)	0.05
E/E’ lateral, Me (IQR)	9.35 (7.9, 10.8)	9.3 (7.8, 10)	10 (7.8, 12)	0.35
E/e’ medial, Me (IQR)	13 (10, 16.1)	13 (10.6, 15.1)	13.13 (8, 28.3)	0.79
E/A, Me (IQR)	0.84 (0.6, 1.5)	1 (0.6, 1.5)	0.8 (0.4, 1.8)	0.44
SARC-F ≥ 4, n (%)	31 (79.5)	23 (76.7)	8 (88.9)	0.65
EPIC category—inactive, n (%)	14 (50)	15 (51.7)	5 (55.6)	0.21
EPIC category—moderately inactive, n (%)	13 (34.2)	10 (34.5)	3 (33.3)	0.21
EPIC category—moderately active, n (%)	4 (10.5)	4 (13.8)	0 (0)	0.21
EPIC category—active, n (%)	1 (2.6)	0 (0)	1 (11.1)	0.21
EPIC category—active and moderately active, n (%)	5 (13.2)	4 (13.8)	1 (11.1)	1.00
Falls during last year, n (%)	23 (59)	18 (60)	5 (55.6)	1.00
MNA-Sf 12–14, n (%)	17 (43.6)	14 (46.7)	3 (33.3)	0.75
MNA-Sf 8–11, n (%)	19 (48.7)	14 (46.7)	5 (55.6)	0.75
MNA-Sf 0–7, n (%)	3 (7.7)	2 (6.7)	1 (11.1)	0.75
MNA-Sf, Me (IQR)	11 (10, 13)	11 (10, 13)	11 (8, 12.5)	0.50
Barthel index, Me (IQR)	90 (70, 95)	90 (70, 95)	90 (72.5, 100)	0.44
I-ADL, Me (IQR)	7 (4, 9)	6 (4, 8)	7 (5, 10)	0.35
Norton, Me (IQR)	17 (16, 18)	17 (16, 18)	18 (16.5, 19.5)	0.38
GDS, Me (IQR)	5 (3, 9)	5 (3, 9)	7 (1.5, 10.5)	0.81
Blessed, Me (IQR)	6 (2, 12)	6 (2, 12.25)	4 (4, 11)	0.96
POMA, Me (IQR)	18 (15, 22)	18 (15.3, 23)	16 (13, 22)	0.34
Morbidity count, n, Me (IQR)	6 (5, 8)	6 (4, 7)	7 (5.5, 8)	0.15
Multimorbidity, n (%)	30 (76.9)	22 (73.3)	8 (88.9)	0.65
Hypertension, n (%)	30 (76.9)	25 (83.3)	5 (55.6)	0.17
Orthostatic hypotension, n (%)	12 (30.8)	7 (23.3)	5 (55.6)	0.10
Diabetes mellitus, n (%)	13 (33.3)	10 (33.3)	3 (33.3)	1.00
Carcinoma history, n (%)	2 (5.1)	2 (6.7)	0 (0)	1.00
Chronic obstructive pulmonary disease, n (%)	10 (25.6)	9 (30)	1 (11.1)	0.40
Myocardial infarction history, n (%)	6 (15.4)	2 (6.7)	4 (44.4)	0.02
Ischemic heart disease, n (%)	13 (33.3)	8 (26.7)	5 (55.6)	0.13
Asthma, n (%)	3 (7.7)	1 (3.3)	2 (22.2)	0.13
Arthritis, n (%)	3 (7.7)	2 (6.7)	1 (11.1)	0.56
Osteoporosis, n (%)	12 (30.8)	10 (33.3)	2 (22.2)	0.69
Stroke history, n (%)	4 (10.3)	3 (10)	1 (11.1)	1.00
Chronic kidney disease, n (%)	12 (30.8)	9 (30)	3 (33.3)	1.00
Atrial fibrillation, n (%)	26 (66.7)	19 (63.3)	7 (77.8)	0.69
COVID-19 history, n (%)	2 (5.1)	1 (3.3)	1 (11.1)	0.41
Peripheral arterial disease, n (%)	3 (7.7)	2 (6.7)	1 (11.1)	0.56
Valvular heart disease, n (%)	14 (35.9)	11 (36.7)	3 (33.3)	1.00
Parkinson’s disease, n (%)	4 (10.3)	4 (13.3)	0 (0)	0.56
Dementia, n (%)	16 (41)	13 (43.3)	3 (33.3)	0.71
Depression, n (%)	19 (48.7)	13 (43.3)	6 (66.7)	0.27
Urinary incontinence, n (%)	1 (2.6)	1 (3.3)	0 (0)	1.00
Medication count, n, Me (IQR)	9 (7, 14)	10 (6.8, 14.5)	9 (7.5, 12.5)	0.81
Polypharmacy, n (%)	35 (89.7)	27 (90)	8 (88.9)	1.00
ACE-I, n (%)	14 (35.9)	11 (36.7)	3 (33.3)	1.00
ARB, n (%)	8 (20.5)	6 (20)	2 (22.2)	1.00
Beta-blockers, n (%)	28 (71.8)	21 (70)	7 (77.8)	1.00
Alpha1-blockers, n (%)	7 (17.9)	4 (13.3)	3 (33.3)	0.32
Loop diuretics, n (%)	25 (64.1)	19 (63.3)	6 (66.7)	1.00
MRA, n (%)	16 (41.0)	14 (46.7)	2 (22.2)	0.26
Thiazide diuretics, n (%)	5 (12.8)	5 (16.2)	0 (0)	0.32
Calcium channel blockers, n (%)	11 (28.2)	9 (30)	2 (22.2)	1.00
Statin, n (%)	11 (28.2)	7 (23.3)	4 (44.4)	0.24
Fibrate, n (%)	1 (2.6)	1 (3.3)	0 (0)	1.00
VKA, n (%)	3 (7.7)	3 (10)	0 (0)	1.00
NOAC, n (%)	22 (56.4)	17 (56.7)	5 (55.6)	1.00
Digoxin, n (%)	5 (12.8)	4 (13.3)	1 (11.1)	1.00
Steroids, n (%)	1 (2.6)	1 (3.3)	0 (0)	1.00
Neuroleptics, n (%)	5 (12.8)	3 (10)	2 (22.2)	0.57
Memantine, n (%)	1 (2.6)	1 (3.3)	0 (0)	1.00
Donepezil, n (%)	3 (7.7)	2 (6.7)	1 (11.1)	0.56
Rivastigmine, n (%)	1 (2.6)	1 (3.3)	0 (0)	1.00
Benzodiazepine, n (%)	5 (12.8)	4 (13.3)	1 (11.1)	1.00
SSRI, n (%)	16 (41.0)	14 (46.7)	2 (22.2)	0.26
NSAID, n (%)	6 (15.4)	5 (16.7)	1 (11.1)	1.00

NYHA—New York Heart Association; BMI—body mass index; TUG—Timed Up and Go; 4MWT—4.57 m walk test; SMM—skeletal muscle mass; SMI—skeletal muscle index; ALM—appendicular lean mass; MNA-Sf—Mini Nutritional Assessment—Short Form; I-ADL—instrumental activities of daily living; GDS—Geriatric Depression Scale; POMA—Performance Oriented Mobility Assessment; MSTN—myostatin; CREA—creatinine; eGFR—estimated glomerular filtration rate; CRP—C-reactive protein; NT-proBNP—N-terminal prohormone of brain natriuretic peptide; LVEF—left ventricular ejection fraction; LVIDD—left ventricular internal diastolic diameter; LAVI—left atrial volume index; LVMI—left ventricular mass index; ACE-I—angiotensin-converting enzyme inhibitors; ARB—angiotensin receptor blockers; MRA—mineralocorticoid receptor antagonists; VKA—vitamin K antagonists; NOAC—non-Vitamin K antagonist oral anticoagulants; SSRI—selective serotonin reuptake inhibitors; NSAID—non-steroidal anti-inflammatory drugs.
